# Multi-branch Convolutional Neural Network for Identification of Small Non-coding RNA genomic loci

**DOI:** 10.1038/s41598-020-66454-3

**Published:** 2020-06-11

**Authors:** Georgios K. Georgakilas, Andrea Grioni, Konstantinos G. Liakos, Eliska Chalupova, Fotis C. Plessas, Panagiotis Alexiou

**Affiliations:** 1 Central European Institute of Technology, Brno, Czech Republic; 2 Faculty of Science, National Centre for Biomolecular Research, Masaryk University, Brno, Czech Republic; 3 Department of Electrical and Computer Engineering, School of Engineering, University of Thessaly, Volos, Greece

**Keywords:** Computational biology and bioinformatics, Machine learning, Transcriptomics

## Abstract

Genomic regions that encode small RNA genes exhibit characteristic patterns in their sequence, secondary structure, and evolutionary conservation. Convolutional Neural Networks are a family of algorithms that can classify data based on learned patterns. Here we present MuStARD an application of Convolutional Neural Networks that can learn patterns associated with user-defined sets of genomic regions, and scan large genomic areas for novel regions exhibiting similar characteristics. We demonstrate that MuStARD is a generic method that can be trained on different classes of human small RNA genomic loci, without need for domain specific knowledge, due to the automated feature and background selection processes built into the model. We also demonstrate the ability of MuStARD for inter-species identification of functional elements by predicting mouse small RNAs (pre-miRNAs and snoRNAs) using models trained on the human genome. MuStARD can be used to filter small RNA-Seq datasets for identification of novel small RNA loci, intra- and inter- species, as demonstrated in three use cases of human, mouse, and fly pre-miRNA prediction. MuStARD is easy to deploy and extend to a variety of genomic classification questions. Code and trained models are freely available at gitlab.com/RBP_Bioinformatics/mustard.

## Introduction

Since the human genome was first sequenced about two decades ago^1^, our understanding of regulatory and non-coding elements in humans, and other organisms, has been steadily increasing with the identification and cataloguing of a variety of encoded molecule and regulatory region classes^2^. Several small non-coding RNA molecule families such as microRNA (miRNA), small nucleolar RNA (snoRNA), small nuclear RNA (snRNA), piwi-interacting RNA (piRNA), short hairpin RNA (shRNA), small interfering RNA (siRNA), promoter-associated short RNAs (PASRs), termini-associated short RNAs (TASRs)^3,4^, transcription initiation RNAs (tiRNAs)^5^, and others, now populate the functional expression map of known genomes. The plethora of functional small non-coding RNA classes supports the idea of a highly interconnected transcriptomic landscape and highlights the necessity of computational approaches that can effectively identify them against the enormous background variability of eukaryotic genomes. Along with our deeper understanding of well-established organisms, the total number of sequenced genomes has been increasing hand in hand with fast pace. NCBI currently lists just over 7,000 eukaryotic sequenced genomes, of which almost 50 have fully assembled genomes, and approximately 1,000 have some assembled chromosomes. The experimental annotation of newly sequenced genomes is a much slower and piecemeal process that benefits greatly from the availability of computational techniques that can guide and assist the annotation.

Computational methods for genomic annotation have a history at least as long as full genome sequencing, with computational identification of exons and protein coding genes^6^ starting in parallel with the sequencing of the first human genome. Small non-coding RNAs, with their shorter length, lack of coding three nucleotide periodicity pattern, and often small number of known examples per class, offer a tougher challenge for computational methods. A common approach for in silico identification of putative small non-coding RNA genomic loci has been the use of sequence homology between molecules from well annotated species, such as humans, and the new species in question. These methods, while efficient when homology is high, are bound to preferentially annotate a subset of loci, biased towards extra-conserved molecules. However, a large number of small non-coding RNAs are more evolutionary constrained. For example, an estimated 40% of human miRNAs have developed recently in evolutionary history and can only be found in other primates^7^.

To avoid the constraints and biases of homology based identification of small non-coding RNA loci, there has been a steady development of algorithms that aim at modelling characteristics of a specific class or subclass, and then evaluating proposed regions of a genome for their potential to encode a small non-coding RNA of this class. For example, over thirty computational methods aiming at pre-microRNA identification have been developed to date, with no tool significantly outperforming all others on benchmarked datasets^8^. A large drawback of such methods is their dependence on expert-defined features and background sets, which tend to produce methods that perform well in evaluations closely matching their training biases, but fail to produce robust classification in more realistic conditions, such as when ‘scanning’ a large genomic region. The second large drawback of these methods is that they are, by design, focused on one specific class or subclass of small non-coding RNA molecules. For example, a method tailored for pre-miRNA prediction is not suitable for snoRNA prediction and vice versa. This issue leads to an unbalanced development of methods towards specific families and ignores others that may not be populous or well-researched enough to warrant the attention of in silico method developers. For example, as mentioned above, pre-microRNA prediction is a well-researched field with over thirty computational methods published in the past decade or so, while in contrast snoRNA prediction displays a distinct paucity of options, with methods becoming obsolete and unusable after more than a decade^9,10^ and the rate of identification severely slowing down in new species^11^.

**Figure 1:**
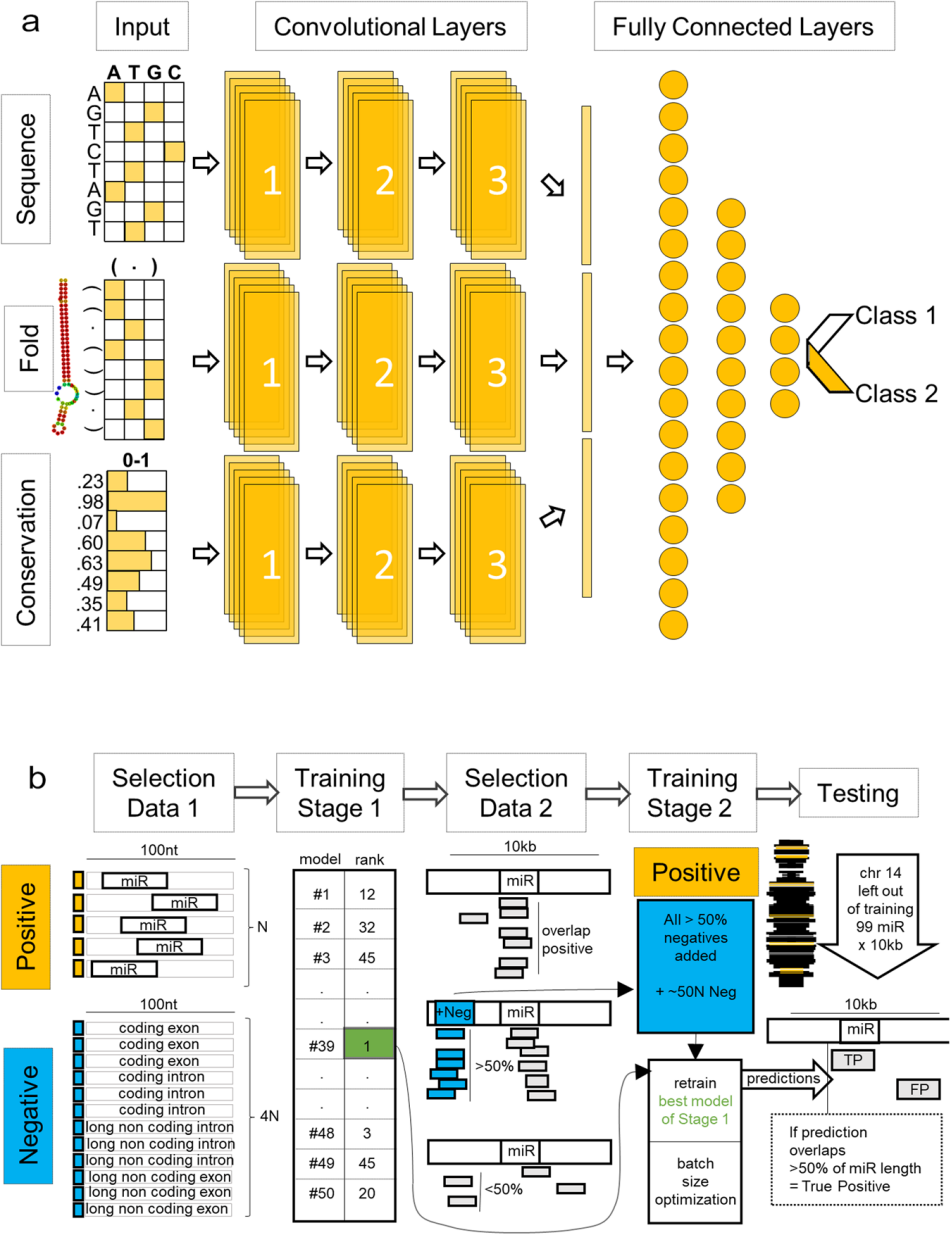
Overview of MuStARD modular architecture and iterative training pipeline. (**a**) MuStARD is able to handle any combination of either raw DNA sequences, RNAfold derived secondary structure and basewise evolutionary conservation from PhyloP. DNA sequences and RNAfold output are one-hot encoded while PhyloP score is not pre-processed. Each feature category is forwarded to a separate ‘branch’ that consists of three convolutional layers. The computations from all branches are concatenated prior to being forwarded to the fully connected part of the network. (**b**) The training pipeline of MuStARD consists of two steps. Initially, pre-miRNA sequences are randomly shuffled to exonic and intronic (protein-coding and lincRNA genes) regions of the genome to extract equal sized negative sequences with 1:4 positive to negative ratio. This process is repeated 50 times to facilitate the training of equal number of models. The performance of each model is assessed based on the test set and all false positives that are supported by at least 25 models are extracted. This set of false positives is added to the negative pool of the best performing model to create an enhanced training set. The enhanced set is used to train the final MuStARD model.

Taking into account the limitations and drawbacks of in silico methods to date, we have decided to approach the problem of small non-coding RNA identification from a different angle. Here we introduce MuStARD (Machine-learning System for Automated RNA Discovery), a flexible Deep Learning framework that utilizes raw sequence, conservation, and folding data to identify genomic loci with similar characteristics of a given set of regions (Fig. 1a). Instead of hundreds of expertly curated features, we employ a Convolutional Neural Network (CNN) Deep Learning (DL) architecture that can identify important characteristics from raw data directly^12^. Rather than biased background training sets, we opted for a novel iterative background selection process that allows the method itself to identify the background ‘hard cases’ for a specific classification task, and preferentially learn how to avoid them. While other tools focus on one class of small non-coding RNAs, we have developed a framework that can be applied, directly out of the box, on any class of genomic loci. We show the power of this methodology by training models that outperform the state of the art for pre-miRNAs and snoRNAs by scanning large genomic regions. We demonstrate the practical use of our method by performing a cross-species prediction using models trained on human data to accurately identify mouse pre-miRNAs and snoRNAs in numbers well above homology searches. Additionally, we applied MuStARD on small RNA-Seq enriched regions and pre-miRNAs that have been removed from miRBase since version 14, to further highlight the usability spectrum of our algorithm. The source code is available at https://gitlab.com/RBP_Bioinformatics/mustard and trained models at https://gitlab.com/RBP_Bioinformatics/mustard_paper.

## Methods

### Network architecture and training scheme

MuStARD is able to handle any combination of either raw DNA sequences, basewise evolutionary conservation and folding data (Fig. 1a). Each feature category is forwarded to a separate ‘branch’ that consists of three convolutional layers and the computations from all branches are concatenated prior to being forwarded to the fully connected part of the network. The training scheme consists of two steps (Fig. 1b). First 50 models are trained in parallel with random background selection. The 50 trained models are used to scan a large region of the genome. From these 50 scans, the ‘hard cases’ where the majority of models detect a false positive are isolated and a new negative set created. The best performing of the 50 models is then used as the starting point to train the final model on the ‘hard cases’ while keeping the same positive set. This final trained model is then evaluated on targets located in chromosomes completely left out of the whole previous process, thus ensuring no cross-contamination.

This process was repeated 6 times to train pre-miRNA detection models composed of different input combinations; raw sequence with secondary structure and conservation (MuStARD-mirSFC model), raw sequence and conservation (MuStARD-mirSC), raw sequence and secondary structure (MuStARD-mirSF), secondary structure and conservation (MuStARD-mirFC), secondary structure only (MuStARD-mirF) and sequence only (MuStARD-mirS). For the combination of raw sequence, secondary structure and conservation, we have trained an additional model after disabling the class weights option in Keras (MuStARD-mirSFC-U model).

The same pipeline was used to create three snoRNA detection models, one for detecting the C/D box snoRNA subspecies (MuStARD-snoSFC-U-CDbox), one for H/ACA box (MuStARD-snoSFC-U-HACAbox) and one for detecting all types of snoRNAs (MuStARD-snoSFC-U).

Detailed information related to the network architecture and training scheme can be found in Supplementary Methods.

### Training sets

Human (GRCh38) and mouse (GRCm38) genomes and corresponding gene and snoRNA annotations were downloaded from Ensembl v93 repository^13^. Fly genome (version 5.32) was downloaded from FlyBase^14^. Pre-miRNA sequences were downloaded from miRBase v22.1^15^. Basewise conservation scores, based on phyloP algorithm, of 99 and 59 vertebrate genomes with human and mouse respectively were downloaded from the UCSC genome repository^16^. For genome scanning tests, targets were extended by + /− 5,000 bp and the resulting regions were merged in the case of strand specific overlaps. The regions were assessed by a moving window of width 100 and step 5. Any prediction overlapping the target by at least 50% was considered a positive. A full explanation of the production of Training Sets, and the Methodology of comparisons can be found in Supplementary Methods. Results of the comparison between MuStARD models using distinct combinations of raw sequence, secondary structure and evolutionary conservation as input, are presented in Supplementary Table 1. The evaluation of the final MuStARD models and comparisons to other state of the art programs can be found in Supplementary Tables 2–7. The performance of MuStARD-mir models in the first training iteration can be found in Supplementary Table 8.

### Software and hardware requirements

MuStARD is developed in python utilizing tensorflow and Keras for the Deep Learning aspect, R for visualizing the performance and Perl for file processing, reformatting and module connectivity. Full list of dependencies can be found on MuStARD’s gitlab page.

MuStARD is able to execute either on CPU or GPU depending on the underlying hardware configuration by taking into advantage tensorflow’s flexibility. The framework has been designed to maintain a minimal memory footprint thus allowing the execution even on personal computers. Running time heavily depends on input dimensionality, number of instances in the training set, learning rate and GPU availability. On a GPU (NVIDIA GeForce GTX 1050Ti) it took approximately 5 minutes to train a model on 30,000 positive and negative sequences.

MuStARD operates directly on genomic intervals in BED format, in the cases of both the training and prediction modules. For example, regarding small RNA-Seq datasets, MuStARD does not directly process aligned reads. Instead, users need to provide a bed file with small RNA-Seq enriched regions to be scanned with MuStARD. Essentially, sequencing depth is not crucial as our algorithm works after a ‘peak calling’ step that can be as simple as a bedtools merge command followed by a bedtools coverage filtering.

For the mouse liver small RNA-Seq dataset, we scanned 14,552 intervals (both strands derived from 7,276 peaks) with a total size of 3.9mbp, mean interval size of 268 bp and standard deviation of 50.3 bp. The MuStARD-mirSFC-U model was used with a sliding window step of 10 bp and the total running time was 36 minutes (CPU usage only).

In the case of the fly embryo dataset, we scanned 1,638 intervals (819 peaks) with 526 kb total size, 321 bp mean size with standard deviation of 172 bp. We used MuStARD-mirSF model with a sliding window step of 10 bp and total running time of 3.8 minutes.

The average running time of MuStARD per peak, based on the mir-SFC-U model, was 0.17 seconds, while based on the mir-SF model was 0.13 seconds. Running times were normalized on 321 bp average interval size.

## Results

### Training of convolutional neural network model

We compared the performance of MuStARD on all combinations of input data for the pre-miRNA prediction dataset (Supplementary Table 1). As expected, scanning test sequences with various models shows that models including a higher number of meaningful input data branches perform better in retrieval of pre-miRNAs. The model trained on secondary structure and conservation was the best performing two-input model. This result aligns with the identification of pre-miRNA hairpins by the Microprocessor complex during miRNA biogenesis primarily by characteristics of their secondary structure rather than sequence^17^ and the fact that pre-miRNAs have highly conserved regions corresponding to the mature miRNA sequences. Surprisingly, the non-balanced model (MuStARD-mirSFC-U) performs best out of all model combinations including the balanced three input model. Since MuStARD-mirSFC-U outperforms all other models, we will only report results for this model in the following evaluations. For snoRNAs, the equivalent best performing model is MuStARD-snoSFC-U. Detailed explanation of the training scheme can be found in Supplementary Methods.

### Identification of *homo sapiens* pre-miRNA genomic loci

While training MuStARD models, we left-out the entirety of randomly selected chromosome 14 as a final evaluation set that could be fairly used to benchmark MuStARD’s performance against the current state of the art in pre-miRNA prediction. The question of accurate pre-miRNA prediction has been thoroughly researched since there are currently over 30 published pre-miRNA prediction algorithms indexed in the OMICtools^18^ repository. The majority of these studies could not be coerced to run on our benchmarking dataset (see Supplementary Methods for details). We managed to run and evaluate five state of the art programs: HuntMi^19^, microPred^20^, MiPred^21^, miRBoost^22^ and triplet-SVM^23^. A list of algorithms we attempted, but failed, to evaluate can be found along with our code repository. Of these five, only triplet-SVM, MiPred and miRBoost provide probabilities as output scores allowing assessment of their performance on multiple score thresholds. HuntMi and microPred provide fixed output score/labels limiting their performance comparison on a fixed threshold (Supplementary Figure 1, Supplementary Tables 2 and 3). After evaluating all five algorithms on the chromosome 14 evaluation set, we identified MiPred as the overall optimally performing state-of-the-art algorithm, thus for the sake of brevity we will only report direct in depth comparison to MiPred. Table 1 summarizes the performance results from the pre-labelled and scanning chromosome 14 benchmarks.

**Table 1:** Performance results based on the human chromosome 14 pre-labelled and scanning datasets.

Algorithms	Homo Sapiens - chr14 pre-labelled dataset			Homo Sapiens - chr14 scanning dataset		
	Precision	Sensitivity	F1	Precision	Sensitivity	F1
MuStARD-mirSFC-U	0.958	0.522	0.675	0.953	0.424	0.587
MiPred	0.128	0.977	0.226	0.069	1	0.130
miRBoost	0.063	0.840	0.118	0.080	0.898	0.146
HuntMi	0.147	1	0.256	0.070	0.979	0.131
microPred	0.114	0.977	0.205	0.197	1	0.330
triplet-SVM	0.194	0.931	0.321	0.061	0.898	0.115
Random	0.051	0.545	0.094	N/A	N/A	N/A

**Figure 2:**
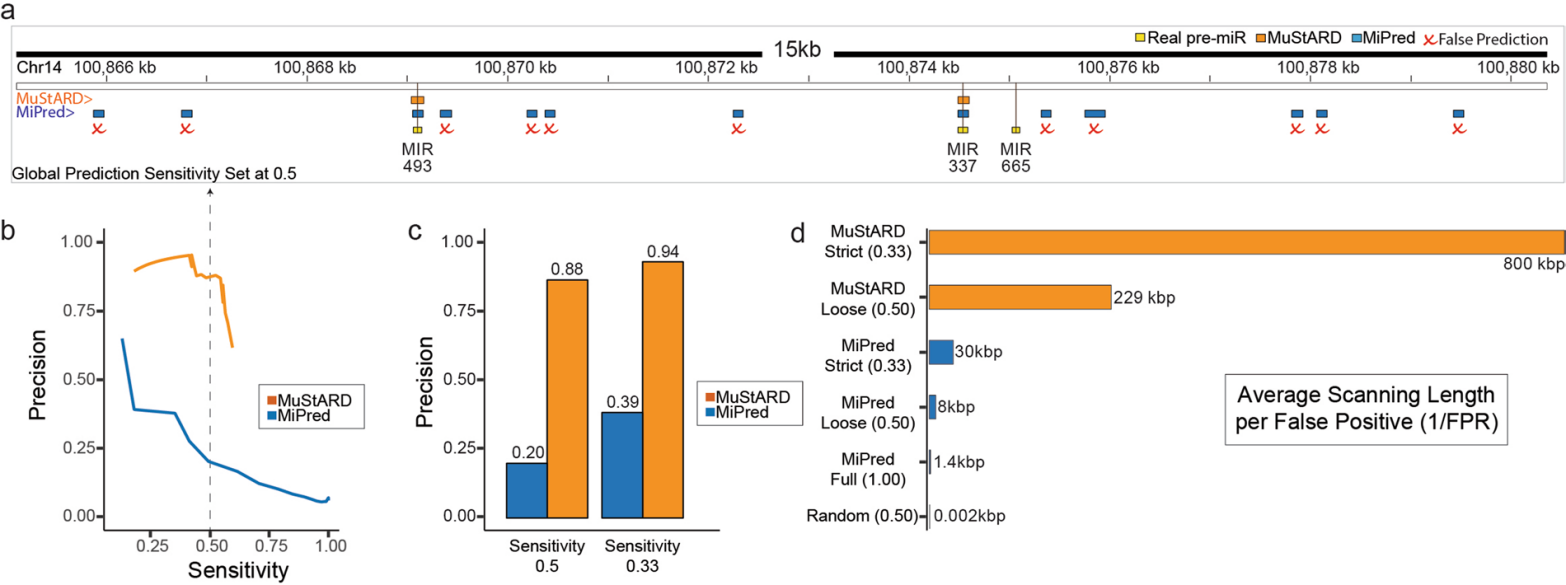
Evaluation of MuStARD human predictions against MiPred, the best performing of state-of-the-art pre-miRNA prediction algorithms. (**a**) Genome browser visualization of each algorithm’s performance on the scanning windows in a 15 kb locus hosting three pre-miRNAs on the left-out chromosome 14. Both evaluated programs have been benchmarked at scores that give sensitivity of 0.5 over the left-out chromosome. MuStARD correctly predicts 2/3 of the annotated pre-miRNAs (in this particular locus), same as MiPred. MuStARD produces no false positive predictions, compared to 11 for MiPred (marked with red x). (**b**) precision-sensitivity curve of MuStARD and MiPred over scanned areas of the left-out chromosome 14. (**c**) Precision of MuStARD and MiPred at loose (sensitivity 0.5) and strict (sensitivity 0.33) thresholds. (**d**) Average length in thousands of base pairs for finding each false positive prediction on the left-out chromosome. Showing MuStARD at strict and loose thresholds, and MiPred at strict, loose, and full (score 0.5 - sensitivity ~1) thresholds, and random prediction (threshold sensitivity 0.5) denoting the worst performing levels an algorithm could achieve.

Both MuStARD and MiPred report predictions with probability scores, and both programs would as default be used at a score threshold of 0.5. However, at that threshold, MiPred produces an inordinate amount of false positives (Supplementary Tables 2 and 3). For fairness of comparison of program precision, we have set a threshold on prediction sensitivity at the point where each program predicts 50% of real pre-miRNAs (Fig. 2a). MuStARD exhibits consistently higher precision for any level of sensitivity (Fig. 2b,c) and at a strict threshold where 33% of real pre-miRNAs can be annotated it produces on average one false positive prediction per 800,000 scanned nucleotides (Fig. 2d) outperforming MiPred by an order of magnitude. Detailed information related to the scanning and static types of evaluation can be found in Supplementary Information.

### Identification of pre-miRNAs from small RNA-Seq data

Our method can scan large genomic regions with unprecedented precision, but would still produce a large number of false positives in a full genome scan of several billion bases. A more realistic experimental and computational approach would be the identification of molecules from small RNA-Seq data. In this type of commonly performed experiment, RNA is isolated and filtered for sizes below a certain threshold, thus removing most mRNAs and long non-coding RNAs. However, there still remain fragments and other artifacts, and the bona fide small RNAs still need to be classified into different classes.

We used the human pre-miRNA trained model of our method to retrieve pre-miRNA predictions from three small RNA-Seq datasets in varying degrees of evolutionary distance from humans. The first dataset consists of human H1 cells, in which we only evaluated 502 small RNA-Seq enriched regions from the left-out chromosome 14. The second dataset comes from mouse liver and we evaluated on 7,276 enriched regions genome-wide. The last datasets is of higher difficulty as it was derived from drosophila melanogaster, an evolutionary distant organism for which conservation information was not readily available. We evaluated our method without the conservation branch (MuStARD-mirSF) on drosophila using the top 819 small RNA-Seq enriched regions (Table 2).

**Table 2:** Performance summary based on the small RNA-Seq datasets from Homo Sapiens, Mus Musculus and Drosophila Melanogaster, at 0.84 score threshold.

		MuStARD-mirSFC-U	MiPred	Expression
Homo Sapiens - small RNA-Seq in H1 cells	Precision	0.750	0.857	1
	Sensitivity	0.500	0.157	0.027
	F1	0.600	0.266	0.054
Mus Musculus - small RNA-Seq in Liver	Precision	0.747	0.512	0.964
	Sensitivity	0.581	0.097	0.065
	F1	0.653	0.163	0.122
Drosophila Melanogaster - small RNA-Seq in Embryo	Precision	0.526	1	0.500
	Sensitivity	0.500	0.023	0.052
	F1	0.512	0.046	0.095

**Figure 3:**
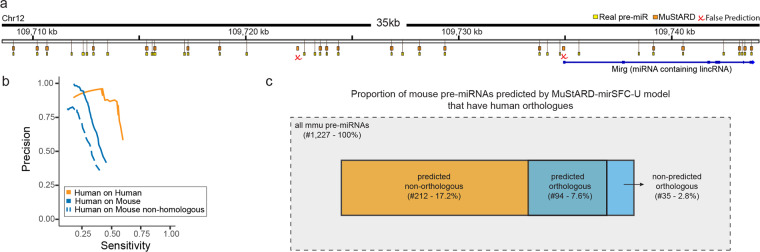
Evaluation of filtering for small RNA-Seq datasets for pre-miRNAs. F1 score per score threshold of the prediction method. MiPred default score threshold is 0.5. We evaluated three datasets: (**a**) human H1 cells, left out chromosome 14. (**b**) mouse liver, whole genome. (**c**) drosophila melanogaster embryo, whole genome. For the drosophila evaluation, vertebrate evolutionary conservation track was not available so the MuStARD-mirSF (sequence, folding) model was used instead.

We have evaluated MuStARD and MiPred using precision/recall curves (Supplementary Figure 2) as well as the F1 measure at multiple score thresholds to gain a spherical view of the algorithms’ performance (Fig. 3). For the precision/recall curves specifically, we have added the ‘naive’ strategy of picking multiple top percentiles of small RNA-Seq enriched loci ranked by decreasing expression level. The ‘naive’ strategy serves as the baseline performance that any Machine Learning algorithm should outperform.

MuStARD outperforms MiPred at every benchmark dataset while keeping a relatively balanced ratio between precision and sensitivity across multiple score thresholds. For example, in order for MiPred to reach high levels of precision (85.7%) in human, it needs to increase the score threshold at a level that reduces the sensitivity below 16% (Supplementary Table 4). MuStARD, on the other hand, is able to maintain sensitivity above 40%, even with a score threshold as high as 0.89 that translates to 82% of precision.

As expected, we notice a decreasing level of MuStARD’s prediction performance with increasing evolutionary distance from our training organism with human (F1 = 0.66, Fig. 3a), mouse (F1 = 0.57, Fig. 3b) and drosophila (F1 = 0.39, Fig. 3c) at 0.5 score threshold (Supplementary Table 4). Our method can narrow down the peaks identified from small RNA-Seq and better prioritize ones that could harbor small RNAs of a specific class. Detailed information related to the small RNA-Seq based strategy can be found in Supplementary Methods.

### Cross-species identification of pre-miRNA genomic loci

**Figure 4:**
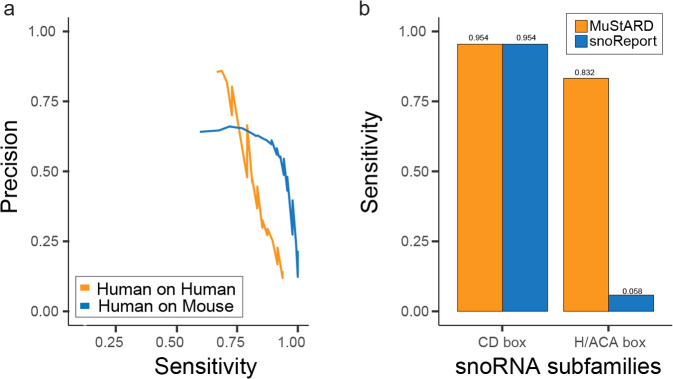
Prediction of mouse pre-miRNAs by the model trained on human. (**a**) Genome browser visualization of MuStARD performance on the scanning of a 35 kb locus hosting 36 pre-miRNAs. MuStARD correctly identifies 20/36 pre-miRNAs with 2 false positives, out of which one falls on the first “exon” of a long non coding RNA Mirg annotated as “miRNA containing lincRNA”. (**b**) Precision-Sensitivity curve of human trained MuStARD predictions on mouse pre-miRNAs. Orange line shows the model prediction on human for reference. Solid blue line shows the prediction on all mouse pre-miRNAs, and dashed blue line shows the prediction on mouse pre-miRNAs without a direct human homologue. (**c**) A visualization of the mouse pre-miRNA evaluation set denoting the number of predicted and non-predicted, orthologous and non-orthologous pre-miRNAs.

Having established a substantial increase in precision for intra-species pre-miRNA prediction we evaluated our model on an inter-species prediction. Briefly, we used the best performing pre-miRNA identification model trained on human data, to scan swathes of the mouse genome (in total ~9.8Mbps) containing 1,227 annotated mouse pre-miRNAs. The inter-species prediction correctly identified pre-miRNAs with a small number of false positives, at a rate of 1/260kbp. Figure 4a shows a browser snapshot of a mouse pre-miRNA cluster locus. As expected, the precision of the inter-species prediction was lower than the intra-species evaluation set (Fig. 4b), and even lower for pre-miRNAs that do not have a human homologue as they have lower levels of conservation which is one of our model’s input branches (Fig. 4c). MuStARD exhibits exceptional levels of generalisation capacity (Supplementary Table 5) identifying correctly a large majority (94/129) of homologous pre-miRNAs and more than double (212) non-homologous pre-miRNAs. Detailed information can be found in Supplementary Methods.

### Evaluation of miRBase retracted pre-miRNAs

In order to evaluate our method on another difficult and realistic task, we mined all pre-miRNAs that were annotated in previous versions of miRBase (version 14 to 22.1) but have since been retracted from it. Extensive details can be found in Supplementary Methods. These are loci that show a close similarity to bona fide microRNAs, enough to be suggested by some experimental method. We evaluated 57 human and 64 mouse pre-miRNAs with our pre-miRNA prediction model trained on the latest human miRBase. Since these retracted pre-miRNAs do not exist in this latest version, our training model has never seen them before. At the 0.5 score threshold (loose) used for the evaluation, MuStARD correctly identified as negative 54/57 (95%) which increased to 56/57 (98%) at 0.85 threshold (strict) for human pre-miRNAs. For mouse pre-miRNAs, still using the human trained model, we correctly retrieved as negative 64/64 (100%) of the targets even at the most loose threshold (Supplementary Table 6).

### Identification of *homo sapiens* sno-RNA loci

Despite its high accuracy on pre-miRNA classification, MuStARD was not specifically developed for pre-miRNA detection. To demonstrate its flexibility we trained models on a completely different class of small non-coding RNAs, small nucleolar RNAs (snoRNAs). SnoRNAs are a class of small RNAs with widely varying structure, sequence, and conservation patterns. We experimentally trained a model on all snoRNAs as well as two additional models for the most populous snoRNAs sub-families, the H/ACA and C/D box. H/ACA box snoRNAs have a secondary structure consisting of hairpins and single stranded regions. In contrast, C/D box snoRNAs have a stem-box structure that is much more variable than H/ACA box. In addition, our ‘all snoRNA’ dataset includes snoRNAs beyond these two sub-families. For the two sub-families we were also able to benchmark against snoReport^24^ a state-of-the-art snoRNA prediction software developed specifically to identify each of these two categories against background (Table 3, Supplementary Table 7). We observe that MuStARD matches snoReport on the Homo Sapiens C/D box training set (Score 0.8, F1: 0.759 vs 0.769), but completely outperforms snoReport in Mus Musculus prediction for both C/D box (Score 0.8, F1: 0.704 vs 0.570) and H/ACA box (Score 0.8, F1: 0.810 vs 0.033). MuStARD also outperforms snoReport at the Homo Sapiens H/ACA box model (Score 0.8, F1: 0.755 vs 0.094). Furthermore, we tested the inter-species capabilities of the MuStARD model, by applying the human-trained snoRNA model to the mouse genome (Supplementary Table 7). These results demonstrate that the MuStARD method is capable of producing well trained models beyond the state of the art without domain knowledge, and even with relatively heterogeneous positive samples (“all snoRNAs”).

**Table 3:** Evaluation of prediction for all snoRNA, and CD-box orH/ACA-box subfamilies separately.

			All snoRNAs	C/D box		H/ACA box	
			MuStARD	MuStARD	snoReport	MuStARD	snoReport
Score Threshold 0.5	Homo Sapiens	Precision	0.545	0.476	0.512	0.705	0.100
		Sensitivity	0.791	0.954	0.954	0.764	0.058
		F1	0.645	0.635	0.666	0.733	0.073
	Mus Musculus	Precision	0.549	0.494	0.332	0.730	0.103
		Sensitivity	0.928	0.969	0.897	0.951	0.146
		F1	0.689	0.654	0.484	0.825	0.120
Score Threshold 0.8	Homo Sapiens	Precision	0.820	0.645	0.666	0.909	0.250
		Sensitivity	0.708	0.954	0.954	0.647	0.058
		F1	0.759	0.769	0.784	0.755	0.094
	Mus Musculus	Precision	0.656	0.580	0.420	0.772	0.055
		Sensitivity	0.769	0.897	0.887	0.853	0.024
		F1	0.708	0.704	0.570	0.810	0.033

## Discussion

We present here a flexible Deep Learning framework that can be used to identify small RNA genomic loci based on the sequence, conservation, and secondary structure characteristics of the class. A model can be easily trained, without any changes on the code, to identify any class of small RNA loci provided enough examples of the class exist. Training of the model does not require expertly curated features specific to the RNA class. In contrast to highly specific methods that rely on extraction of hundreds of features, our method operates directly on raw sequence, conservation scores, and a simple linear folding representation. Despite the simplicity of the inputs, and the generality of the method, it manages to convincingly outperform all state of the art methods that have been each developed and trained on one single class of RNAs specifically.

An important aspect of our method is the ability, for the first time, to scan large genomic regions, or even several thousand sequencing peaks, at an acceptably low false discovery rate. Machine learning methods can only learn variation that is presented to them. When looking for extremely rare events, such as small RNA genomic loci, it becomes evident that large genomic regions will need to be scanned, and the background variation will be enormous. However, most negative loci have extremely low potential of being confused for small RNA loci. A static classification between real loci and randomly selected background is prone to overestimate the predictive power of evaluated methods. Some methods have attempted to create ‘harder’ negative sets by including sequences that have characteristics similar to the predicted class. This approach implies that the researcher already knows the major characteristics of the predicted class, and that these characteristics will remain stable for each training model. In our case neither of these prerequisites were true.

We initially prototyped our method with a small set of negatives, four for each real training example, randomly selected from regions within the coding and intronic regions of mRNAs, and long non-coding RNAs. We quickly realized that while our method could separate between these categories easily, it still produced a large amount of false positives in the more realistic large region scanning evaluation. Training several models using different, but equal, background sets showed us variability in the number and range of identified false positives. However, we noticed that a number of false positives appeared consistently in several of the trained models. These ‘hard cases’ of background variation are the ones that have sequence, conservation, and folding characteristics closest to the real training examples and are thus harder to differentiate. We decided to attempt an iterative enrichment technique for the training background in which ‘hard cases’ that confuse our models consistently are added into the training set for a second round of training. This method achieved a great leap in performance when evaluated in completely independent data. Importantly, this automatic iterative method does not rely on an expert user to select the characteristics of importance. The ‘hard cases’ are identified by the training model itself and will fit to whatever positive set it is training on. The enriched background for pre-miRNA and sno-RNA models is radically different, representing the differences of these classes between them, and allowing the models to be easily and accurately trained on any positive set of small RNA loci.

Using a number of pre-miRNA prediction algorithms for region scanning was time consuming and arduous labor. To calculate hundreds of features on regions spanning less than one percent of the human genome, all other algorithms (with miRBoost being the sole exception) required to group the scanning region into smaller batches of 2000 sequences in order to parallelize the analysis into a computer cluster (MetaCentrum-CERIT). Even so, the computing time for each single batch was approximately 4 days. In contrast, our algorithm was able to scan the mouse benchmark dataset that includes several million base pairs in a few hours on a single CPU.

We have demonstrated that our method can be used for cross-species prediction of small RNAs. As a proof of concept we trained models on human pre-miRNAs and snoRNAs and then identified their counterparts in mouse, a pair of well annotated species that have considerable evolutionary distance. The pre-miRNAs we correctly identified on the mouse genome were enriched in evolutionary conserved pre-miRNAs in human (approximately 30% of our true positive predictions vs 10% of all mouse miRNAs). That said, the majority (70%) of our predicted pre-miRNAs are not homologous to human pre-miRNAs and would not be easily identified by a simple homology search.

The method presented here can be generalized for any class of small RNAs on any species. We chose to highlight two examples (pre-miRNAs and sno-RNAs) that differ radically. Where pre-miRNAs have high levels of conservation and fold into characteristic hairpin structures, sno-RNAs show a much wider size distribution (118.8 mean / 59.1 sd vs 81.9 mean / 16.9 sd) and have a variety of subclasses with variable secondary structure and evolutionary patterns, making their identification harder. Thousands of known pre-miRNA sequences against a few hundred sno-RNAs reduce the size of the training set, adding a level of difficulty to the task. It follows that several methods for pre-miRNA identification have been developed to date, while sno-RNA identification methods have not been developed in the past decade. The need for modern, easy to use, easy to train, methods becomes self-evident, especially for RNA classes with fewer members, for which no new development is performed. It is beyond the scope of this paper to develop models for each class of small RNAs, but using our openly available method researchers can easily produce such models for their own RNAs of interest. MuStARD has been specifically designed to automate this process and facilitate ease-of-use by simplifying the input requirements. Regions of the targeted small RNA class can be loaded as a bed file, and MuStARD handles all pre-processing steps such as sequence and evolutionary conservation extraction as well as secondary structure calculation. Additionally, the iterative training module provides an interface for the automatic selection of background genomic loci that optimally represent the negative set, specifically tailored for the specific small RNA class. MuStARD can be easily applied on any small RNA identification problem that would not be easily identifiable by using older methods. Extensive documentation and tutorials on using MuStARD for novel RNA class predictions are available along with the MuStARD code repository at gitlab.com/RBP_Bioinformatics/mustard.

## Conclusion

To conclude, we have developed a method that is easy to train and deploy for any class of small RNA genomic loci. Using the novel iterative background selection our method can choose the background ‘hard cases’ specific for each training, boosting performance. We show that our method outperforms class specific methods, both in accuracy, and computational performance. We achieved cross species identification of small RNAs beyond homology, and also highlighted a realistic use case in the identification of pre-miRNAs out of small RNA-Seq peaks.

## Supplementary information

Supplementary Table S1

Supplementary Table S2

Supplementary Table S3

Supplementary Table S4

Supplementary Table S5

Supplementary Table S6

Supplementary Table S7

Supplementary Table S8

Supplementary Information
