# Supplementary material for: Multi-branch Convolutional Neural Network for Identification of Small Non-coding RNA genomic loci
Source: Sci Rep. 2020 Jun 11;10:9486. doi: 10.1038/s41598-020-66454-3 (PMC7289789; doi:10.1038/s41598-020-66454-3)
Supplement: Supplementary file 9 — Supplementary Information [file 41598_2020_66454_MOESM9_ESM.pdf]

Supplementary Information

Multi-branch Convolutional Neural Network for Identification of Small Non-coding RNA genomic loci

Georgios K Georgakilas[1], Andrea Grioni[1], Konstantinos G Liakos[3], Eliska Malanikova[2], Fotis C Plessas[3] and Panagiotis Alexiou[1,*].

1. Central European Institute of Technology, Brno, Czech Republic
2. Faculty of Science, National Centre for Biomolecular Research, Masaryk University, Brno, Czech Republic
3. Department of Electrical and Computer Engineering, School of Engineering, University of Thessaly, Volos, Greece

* Correspondence should be addressed to Panagiotis Alexiou.

Supplementary Methods

Dataset collection and preprocessing

Homo sapiens (GRCh38) and Mus musculus (GRCm38) genomes as well as the corresponding gene annotations were downloaded from Ensembl v93 repository[1]. Human gene annotation was filtered to only include genes exhibiting a protein-coding or lincRNA biotype. The exons of human protein-coding and lincRNA genes were combined to produce two disjoint data sets; parts of the genome that correspond to exons and loci that are represented by introns. A separate collection was created by selecting regions marked with the snoRNA biotype for both mouse and human. Drosophila melanogaster genome (version 5.32) was downloaded from FlyBase[2].

Homo sapiens, Mus musculus and Drosophila melanogaster pre-miRNAs were downloaded from a spectrum of miRBase releases, ranging from version 14 to 22.1[3]. Every miRBase release hosts detailed metadata regarding newly added miRNAs, changes in nomenclature and, most importantly, sequences that have been falsely annotated as miRNAs in the previous version. After parsing all relevant files between versions 14 and 22.1, we created a set of 57 and 64 discontinued pre-miRNAs, in human and mouse respectively (Supplementary Table 6). These sequences facilitated the evaluation of MuStARD presented in section "Evaluation of miRBase retracted pre-miRNAs". LiftOver tools from UCSC was used to match genomic coordinates from older to the latest assemblies, wherever needed. Human pre-miRNAs from version 22.1 were filtered based on the experimentally validated information provided in miRBase to keep only high-quality sequences for training the MuStARD models presented in this study.

Basewise conservation scores, based on phyloP algorithm[4], of 99 and 59 vertebrate genomes with human and mouse respectively were downloaded from the UCSC genome repository[5].

MuStARD training module architecture

Aim of MuStARD is to provide a highly flexible, feature-agnostic computational framework that can be applied in a plethora of problems in Biology, providing state-of-the-art performance while at the same time having minimal input requirements. To this end, MuStARD has been specifically designed to follow a modular architecture where each module carries out different functionalities that can be run and assessed independently and/or in parallel (Figure 1a). The framework is implemented using python for the deep learning aspect, R for the majority of meta-analyses and plotting, and perl for general purpose file filtering, formatting and module connectivity. Users only need to provide bed formatted files as input and the appropriate genome assembly files as well as the wiggle formatted PhyloP evolutionary conservation score, typically derived from UCSC repository.

The training module of MuStARD is composed of a convolutional architecture based on tensorflow and Keras functional API. More advanced users can directly add or remove parts of the architecture according to the problem at hand. For the purposes of this study, the chosen architecture consists of 3 convolutional branches that can be dynamically added, removed and combined in multiple ways based on the properties of the corresponding use-case. These branches depict distinct 'agents' that are able to model distinct input information such as raw

DNA sequence, RNA secondary structure and evolutionary conservation. Subsequently, the outputs of the convolutional branches are flattened, concatenated and forwarded to the dense part of the architecture that produces the final prediction scores (or in other words, the per class probabilities). In every layer output, dropout and batch normalization regularization techniques are applied to improve the generalisation capacity of the network.

Regardless of the chosen network architecture, hyperparameters are known to be notoriously hard to optimize and depending on the complexity of the input, small changes in the hyperparameter selection can greatly affect the results. The training module has been designed to incorporate a grid-search type of approach for finding the optimal combination of hyperparameters. We have chosen to apply grid-search over 4 hyperparameters, the ones that based on our experience are able to greatly affect the results; batch size, learning rate, dropout rate and number of filters in the convolutional layers. Users can freely remove or add hyperparameters into the grid-search process and most importantly adjust the network architecture according to their needs. Each model trained over a different combination of hyperparameters is saved in a separate directory alongside train/validation accuracy/loss plots and a detailed log of the performance in each epoch. This allows users to find the exact combination of hyperparameters that produces the optimal training.

Unless stated otherwise, in all use-cases presented in this study, each convolutional branch consists of 3 convolutional layers. The first convolutional layer in the raw DNA sequence processing branch uses a filter size of 16 nucleotides with stride 1 and no padding, the second layer uses a filter size of 12 and the third layer a filter size of 8. The first convolutional layer in the RNAfold processing branch uses a filter size of 30 nucleotides with stride 1 and no padding, the second layer uses a filter size of 20 and the third layer a filter size of 10. The first convolutional layer in the evolutionary conservation processing branch uses a filter size of 20 nucleotides with stride 1 and no padding, the second layer uses a filter size of 15 and the third layer a filter size of 10. The outputs of the convolutional branches are flattened and concatenated before being forwarded to the dense part of the network that includes 3 layers of 100, 75 and 50 nodes respectively. All layers use leaky ReLu activation except the final prediction layer that uses the softmax function. The chosen optimizer is SGD with Nesterov momentum set at 0.9. All models were trained over 600 epochs after enabling early stopping with patience set at 40 and delta at 0 with a learning rate of 10^{-4}. The code is accompanied with a rich documentation and examples of how to edit parts of the architecture and train new or test existing models.

MuStARD prediction module

The prediction module of MuStARD framework has been explicitly designed to facilitate both long region scanning and static assessment of specific loci. In the case of long region scanning, users are able to select the appropriate parameters such as the window size (it should match with the training window size), sliding step and the model that will be used for scoring each window. The framework includes standalone code for generating bedGraph tracks that can facilitate the visualization of results in any genome browser as well as code for creating ‘hotspots’ of positive predictions and for evaluating the results based on custom tracks and/or annotations. In the case of static assessment of specific loci, the prediction module provides a

bed formatted file that includes the MuStARD score of each region.

Learning process of pre-miRNA detection MuStARD models

As described in the previous sections, the training of the pre-miRNA recognition model was based on experimentally verified human precursor sequences from miRBase v22.1. Only pre-miRNAs with size less than 100bp were used to form the positive set resulting in 579 sequences that were separated into the necessary training (484 hairpins corresponding to all chromosomes except 2, 3 and 14) and validation (51 hairpins corresponding to chromosomes 2 and 3) sets required by DL frameworks for the training process. 99 hairpins from chromosome 14 (no experimental validation evidence or size distinction) were completely left out from the training procedure and were utilized for testing the performance of pre-miRNA detection algorithms.

The initial negative set was formulated with bedtools v2.27.0[6] 'shuffle' mode using the positive set on the exon/intron genomic segments described in a previous section (Figure 1b). For each positive instance, 4 equally sized negatives were randomly selected from protein- and non-coding exonic as well as intronic regions, 1 for every category. This process was repeated 50 times in total creating 50 different sets that were used to train an equal amount of distinct preliminary models. Hyperparameters were fixed at 256 batch size, 0.2 dropout rate, 0.0001 learning rate and 80/40/20 number of filters in the 3 convolutional layers of each branch and the class weight option in Keras was enabled. Based on this repetitive negative shuffling configuration we ensured that a reasonable balance between training time as well approximating sequence and evolutionary conservation variation in background (or in other words, non-precursor genomic loci) was maintained. Instances of the training set located in chromosomes 2 and 3 were used for validation, regions in chromosome 14 were left out of the training process and all remaining loci were used for training. One of our objectives was to optimize the genome scanning process. The majority of existing algorithms utilize positive sequences that are fixated around the center of pre-miRNAs. However, in genome scanning scenarios there will always be instances in which part or the whole hairpin sequence will not be located in the center of the scanning window. This phenomenon might heavily affect the secondary structure of the RNA sequence corresponding to each window and therefore the generalisation capacity of the model. To overcome this problem, the MuStARD training module has been equipped with an optional 'augmentation' feature that generates copies of the input instances after randomly placing positive or negative sequences within the 100nt sequence. For the purposes of this study, the number of augmented instances for every input sequence was 5.

If the combination of using intronic/exonic regions as a background sequence pool and the 1:4 positive to negative ratio was enough to fully capture the non-precursor sequence variation in the 3 input feature space (raw DNA sequence, secondary structure and basewise evolutionary conservation) then a near perfect performance in terms of both precision and sensitivity would be achieved in a scenario where all 50 preliminary models are used to scan the genome for predicting pre-miRNA sequences. To test this hypothesis all human pre-miRNAs were extended by +/- 5,000bp and the resulting regions were merged in the case of strand specific overlaps. Both strands of the merged loci were scanned with all 50 preliminary models using a

window of 100bp and a stride of 10bp. This resulted in a benchmark dataset of 33.2 million bp divided into 3.2 million overlapping 100bp windows. For each model, out of the 3.2 million windows only those exhibiting a score above 0.5 were retained to form ‘hotspots’ of positively predicted regions after merging cases of strand specific overlaps. These regions were subsequently cross checked with the annotated pre-miRNAs to extract performance metrics for every preliminary model.

False positive predictions based on a 0.5 score threshold were kept only if they were supported by 25 out of 50 preliminary models and did not overlap with any negative instance used to train these models. The resulting 23,750 false positive loci were added to the negative dataset of the best performing preliminary model. These false positives represent regions of the genome that were not captured by the process of ‘shuffling’ positive instances to exonic/intronic loci and exhibit feature characteristics that are more similar to positive than the initial negative sequences. This process assisted in establishing an enhanced set that was used to train the final pre-miRNA detection model that was selected through performing a hyperparameter space grid-search over the batch size and the Keras option of training with/without class weights. The class weights option in Keras enables the equal contribution of all classes during the training of unbalanced datasets. The remaining hyperparameters were not changed.

This process was repeated 6 times to train (Supplementary Table 1), with the Keras class weights option enabled, an equal number of distinct MuStARD pre-miRNA detection models composed of different input combinations; raw sequence with secondary structure and conservation (MuStARD-mirSFC model), raw sequence and conservation (MuStARD-mirSC), raw sequence and secondary structure (MuStARD-mirSF), secondary structure and conservation (MuStARD-mirFC), secondary structure only (MuStARD-mirF) and sequence only (MuStARD-mirS). For the combination of raw sequence, secondary structure and conservation, we have trained an additional model after disabling the class weights option in Keras (MuStARD-mirSFC-U model). For MuStARD-mirSFC model, the optimal (balance between precision and sensitivity) batch size was 1024, 256 for MuStARD-mirSFC-U, 1024 for MuStARD-mirSC, 256 for MuStARD-mirSF and MuStARD-mirFC, 512 for MuStARD-mirF and MuStARD-mirS. The procedure for evaluating the performance of each model is described in the following section.

For the purposes of snoRNA prediction, the same pipeline was used to create three MuStARD snoRNA detection models, one for detecting the C/D box snoRNA subspecies (MuStARD-snoSFC-U-CDbox), one for H/ACA box (MuStARD-snoSFC-U-HACAbox) and one for detecting all types of snoRNAs (MuStARD-snoSFC-U). For training the MuStARD-snoSFC-U-CDbox model we removed CD box snoRNAs with size more than 130nt. For the validation set we used 28 sequences originating from chromosomes 3, 4, 5, 20, 21 and 22. For the training set we utilized 208 sequences located in the remaining chromosomes except 22 snoRNAs from chromosomes 1 and 2 (no size filtering for the creation of this set) that were used for testing the performance of snoRNA prediction algorithms. For training the MuStARD-snoSFC-U-HACAbox model we removed H/ACA box snoRNAs with size more than 150nt. For the validation set we used 29 snoRNAs originating from chromosomes 2, 3, 4, 20, 21 and 22. For the training set we utilized 94 sequences located in the remaining chromosomes except 17

snoRNAs from chromosome 1 (no size filtering) that were kept for testing. For training the MuStARD-snoSFC-U model we removed snoRNAs with size more than 150nt. For the validation set we used 42 snoRNAs originating from chromosomes 3, 4, 20, 21 and 22. For the training set we utilized 317 sequences located in the remaining chromosomes except 48 snoRNAs from chromosomes 1 and 2 (no size filtering) that consisted the testing set.

Testing on genomic region scanning data

The process of testing algorithms on a static labelled dataset can often provide misleading results about performance especially in cases of models that have been designed for genome-wide scanning. Genome scanning evaluation strategies are able to unveil interesting aspects about the computational complexity and the time required by algorithms to complete a task. To this end, 99 human pre-miRNAs (no size or quality filtering) located on chromosome 14 were extended by +/- 5,000bp and the resulting regions were merged in the case of strand specific overlaps. Both strands of the merged loci were scanned with all MuStARD's final pre-miRNA detection models (Supplementary Table 1) as well as with existing algorithms (Supplementary Table 2) using a window of 100bp and a stride of 5bp. This resulted in a scanning benchmark dataset of 1 million bp divided into 208,708 overlapping 100bp windows.

Two distinct strategies were employed to assess the performance of each algorithm. In the first approach, each window was assessed independently (method A) while in the second only windows exhibiting a score above 0.5 were retained to form 'hotspots' of positively predicted regions without taking strand into consideration (method B). These regions were subsequently cross checked with the 99 annotated chromosome 14 pre-miRNAs to extract performance metrics in the 0.5-0.9 score range, when possible. In both scenarios, positive predictions were considered true positives (TPs) if they covered at least 50% of the overlapping annotated pre-miRNA's length. HuntMi[7] and microPred[8] algorithms only provide hard labelled results instead of a probabilistic score, therefore they were not included in precision/sensitivity curves. However, to facilitate a fair comparison between all algorithms, performance metrics based on both methods A and B were extracted at a fixed score threshold of 0.5 (Supplementary Tables 2 and 3).

For assessing the performance of MuStARD-snoSFC-U, MuStARD-snoSFC-U-CDbox, MuStARD-snoSFC-U-HACAbox, snoReport-CDbox and snoReport-HACAbox models, human snoRNAs on the test chromosomes (see previous section) were extended by +/- 10kbp and the resulting regions were merged in the case of strand specific overlaps. Both strands of the merged loci were scanned with different window sizes (depending on the model) and a stride of 5bp. For comparing the snoRNA detection models, only method B was applied (Supplementary Table 7).

Method B was also applied to annotated mouse pre-miRNAs and snoRNAs using the relevant MuStARD's SFC-U models and snoReport in the case of snoRNAs (Supplementary Tables 5 and 7).

Testing on labelled data

For the purposes of testing the final pre-miRNA detection model on labelled data and comparing with existing algorithms, all positive and negative instances located on chromosome 14 were used from the enhanced set described in the previous section after removing sequences with size less than 100bp. The total number of positive instances in the test set was 44 and the total number of negatives 893 (Supplementary Table 3).

Application of existing algorithms

There are over 30 pre-miRNA prediction algorithms listed in OMICtools repository. The majority of these studies provide access to the trained models only through web-server interfaces which allow a small number of sequences to be processed at once. Only a handful of studies provide stand-alone implementations that can be downloaded and applied on benchmark datasets locally. However, a small fraction of these implementations are able to be properly executed and provide results.

We only managed to assess the prediction efficiency of HuntMi, microPred, MiPred, triplet-SVM, and MirBoost on our benchmark datasets. HuntMi and microPred tools do not support parallelization, and the average processing time for a sequence of 100nt is 3 minutes. The scanning benchmark sequences were grouped into 100 batches to speed-up the analysis for HuntMi and microPred. Also, microPred random sequence generation parameter setting was set at 500. Each batch was analyzed independently by HuntMi and microPred on virtual machines provided by the MetaCentrum-CESNET supercomputer cluster. MiRBoost's SVM model was re-trained to support probabilistic output using the dataset included in the code repository and parameters 'svm-train -h 0 -c 8.0 -g 0.125 -w1 1 -w-1 1 -b 1'. Then miRBoost was applied on our benchmark dataset with parameters 'miRBoost -d 0.25'. For triplet-SVM, we initially applied RNAfold on the benchmark dataset with parameters 'RNAfold --noPS --noconv --jobs=10' and the output was forwarded to the triplet-SVM perl script with parameters 'triplet_svm_classifier.pl 22' that pre-processes the data and reformats it to libsvm standards. The final triplet-SVM results were obtained using svm-predict with parameters 'svm-predict -b 1'. MiPred and snoReport were applied with default parameters.

Assessing MuStARD's ability to detect non-human-homologous pre-miRNAs in mouse

Mouse hairpins (N=1,227) were derived from miRBase v22.1 and homologous miRNAs (N=129) between mouse and human were retrieved from the Ensembl BioMart hub[9]. Initially, MuStARD true positive predictions were recognized as overlapping with annotated mouse hairpins with bedtools intersect module. Subsequently, non-human-homologous pre-miRNAs were distinguished as the negative intersection between true positive MuStARD predictions and the human-mouse orthologous pre-miRNA list. Bedtools options 'same strandedness' and 'overlaps=0.5' were used in both cases (-s and -f, respectively).

Small RNA-Seq based evaluation of MuStARD and MiPred

Small RNA-Seq datasets from human H1 cells (sample ID ENCFF635LZQ) and mouse liver (sample ID ENCFF175XCP) were downloaded from the ENCODE portal[10]. Small RNA-Seq

dataset from drosophila embryo (sample ID 2971) was downloaded from the modENCODE repository[11]. All samples were downloaded in bam format and were further processed to keep reads with a mapping quality of 255.

Macs2[12] was subsequently utilized with parameters '--nomodel --extsize 50 --keep-dup all -q 0.001 --call-summits' to identify genomic loci enriched in small RNA-Seq signal. Resulting peaks (top 1,000 expressed in drosophila due to overall high noise levels) were extended by 100bp on both sides, to ensure that the whole putative pre-miRNAs will be inside the tested regions. Peaks that overlapped after the extension were merged into a unique region. In human, we only considered regions from chromosome 14 (N=502) that were also intersected with the 99 annotated pre-miRNAs to generate the positive (N=38) and negative (N=464) small RNA-Seq human benchmark. The 7,276 mouse regions were intersected with 1,227 pre-miRNAs, resulting to 473 positives and 6,803 negatives. For drosophila, the 819 regions were intersected with 260 annotated precursors to generate 42 positives and 777 negatives.

The aforementioned regions were scanned (both strands) with MuStARD (mirSFC-U for human/mouse and mirSF for drosophila) and MiPred (Supplementary Table 4). Multiple score cutoffs were applied to create precision/recall and F1 curves. We also applied multiple expression cutoffs (log10 transformation and subsequent scaling between 0 and 1) on the three groups of small RNA-Seq enriched regions to generate a non-Machine-Learning set of predictions that would correspond to the naive strategy of selecting top percentiles of expressed regions as real pre-miRNAs. This baseline 'model' is referred to as Expression.

For the purposes of this evaluation approach, the score threshold upper limit was set to 0.96 or the one corresponding to (TP + FP) >= 1 for populating the supplementary tables and (TP + FP) >= 10 for making the figures.

Software and hardware requirements

MuStARD is developed in Python for the Deep Learning aspect, R for visualizing the performance and Perl for file processing, reformatting and module connectivity. Full list of dependencies can be found on MuStARD's gitlab page.

MuStARD is able to execute either on CPU or GPU depending on the underlying hardware configuration by taking into advantage tensorflow's flexibility. The framework has been designed to maintain a minimal memory footprint thus allowing the execution even on personal computers. Running time heavily depends on input dimensionality, number of instances in the training set, learning rate and GPU availability.

Supplementary Figures

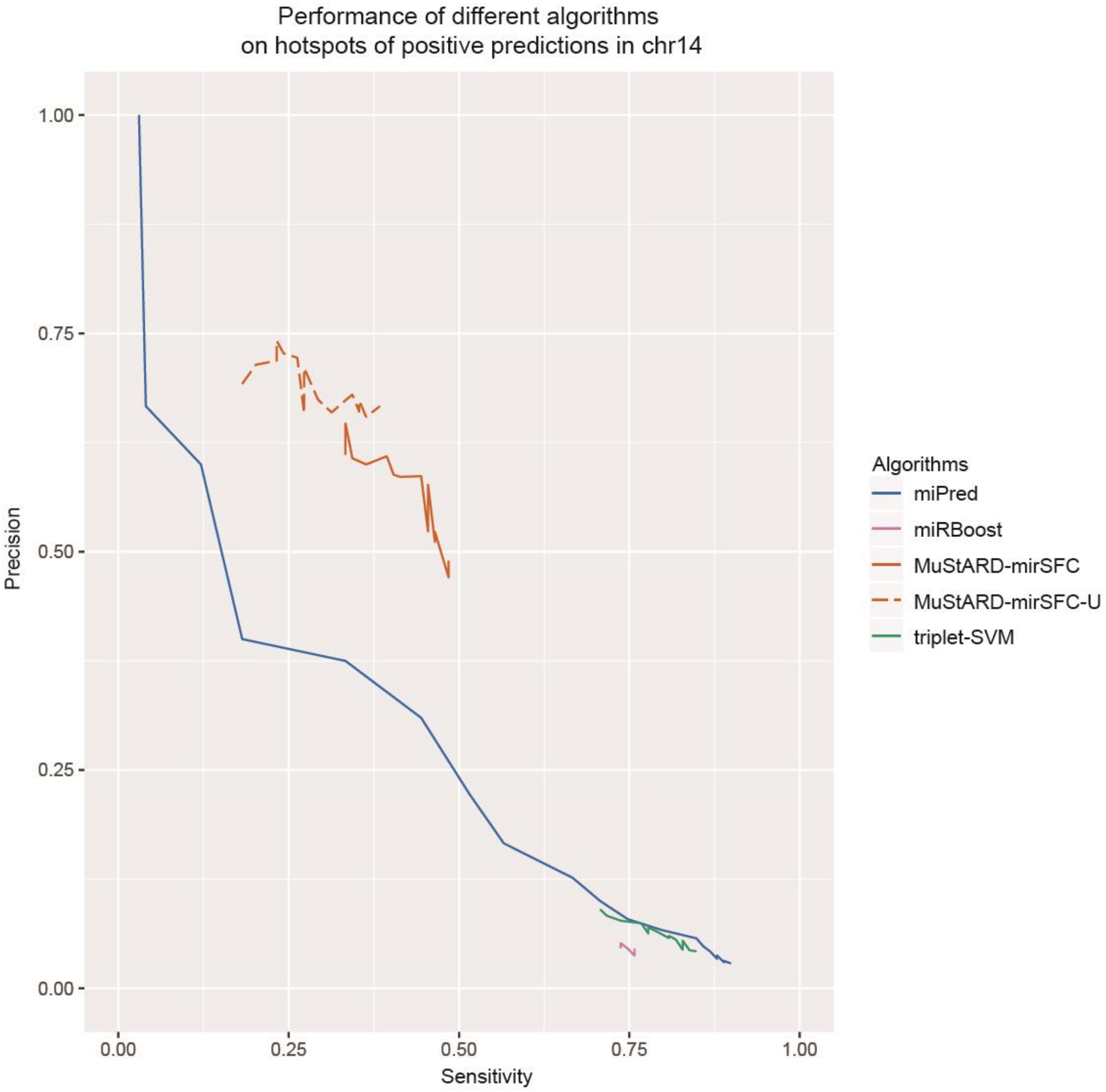

Supplementary Figure 1. Precision/Sensitivity curves comparing the performance of MuStARD-mirSFC (unbalanced model denoted with -U extension) and existing algorithms on the benchmarking dataset of annotated human pre-miRNAs in chromosome 14. HuntMi and microPred algorithms were not included in this graph since they do not provide a continuous score for their predictions. Detailed information related to the performance of all algorithms on this dataset can be found in Supplementary Table 2.

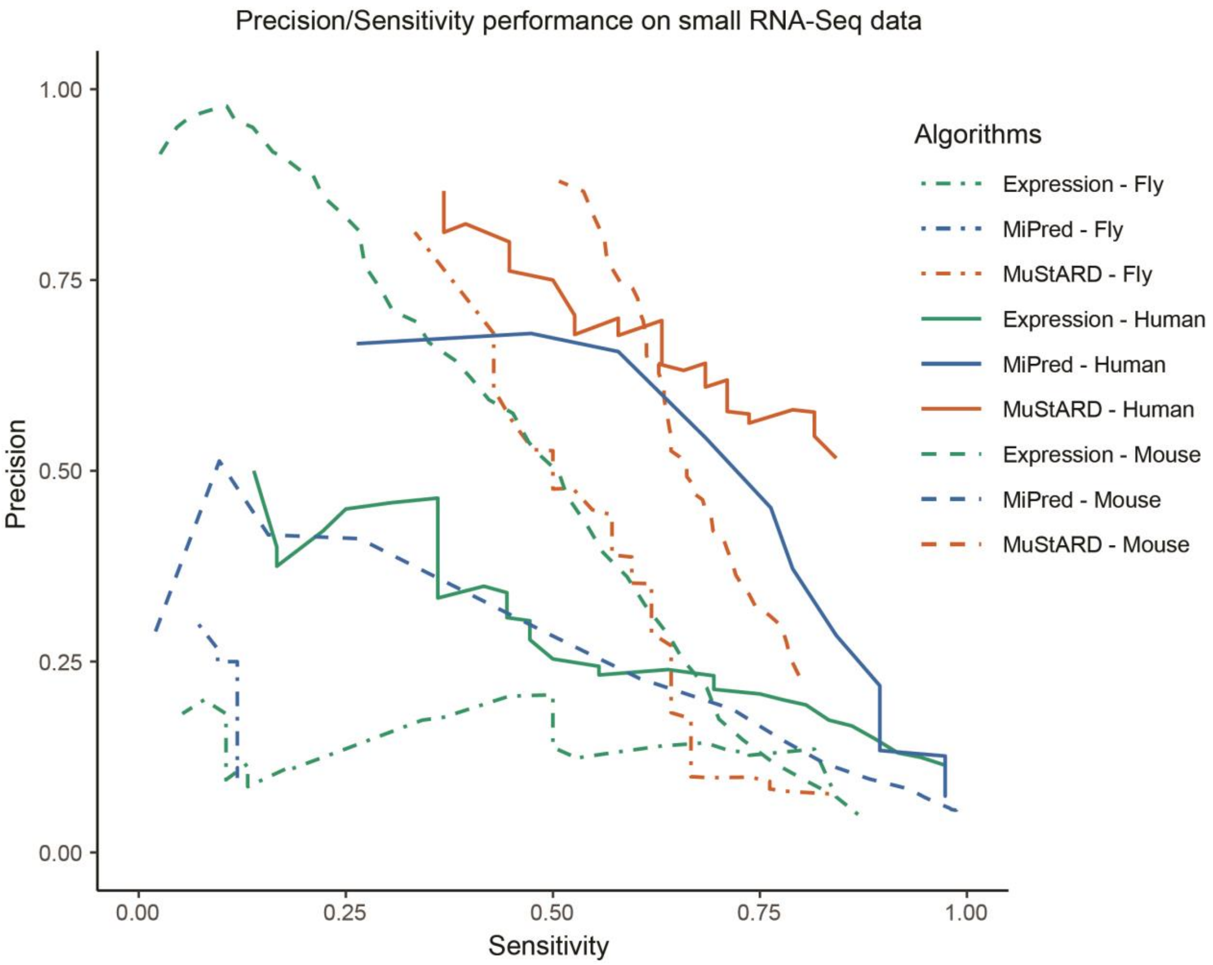

Supplementary Figure 2. Precision/Sensitivity curves depicting the performance of various approaches for pre-miRNA detection on small RNA-Seq enriched regions in *Homo sapiens* (solid line), *Mus musculus* (dashed line) and *Drosophila melanogaster* (dot-dashed line). MuStARD-mirSFC (human and mouse) and -mirSF (fly) models are shown in orange, MiPred in blue and the Expression based approach (see Supplementary Methods) in green. Additional details on the performance metrics can also be found in Supplementary Table 4.

References

1. Zerbino, D. R. *et al.* Ensembl 2018. *Nucleic Acids Res.* **46**, D754–D761 (2018).
2. Thurmond, J. *et al.* FlyBase 2.0: the next generation. *Nucleic Acids Res.* **47**, D759–D765 (2019).
3. Kozomara, A., Birgaoanu, M. & Griffiths-Jones, S. miRBase: from microRNA sequences to function. *Nucleic Acids Res.* (2018) doi:10.1093/nar/gky1141.
4. Cooper, G. M. *et al.* Distribution and intensity of constraint in mammalian genomic sequence. *Genome Res.* **15**, 901–913 (2005).
5. Karolchik, D. *et al.* The UCSC Table Browser data retrieval tool. *Nucleic Acids Res.* **32**, D493–6 (2004).
6. Quinlan, A. R. & Hall, I. M. BEDTools: a flexible suite of utilities for comparing genomic features. *Bioinformatics* **26**, 841–842 (2010).
7. Gudyś, A., Szcześniak, M. W., Sikora, M. & Makałowska, I. HuntMi: an efficient and taxon-specific approach in pre-miRNA identification. *BMC Bioinformatics* **14**, 83 (2013).
8. Batuwita, R. & Palade, V. microPred: effective classification of pre-miRNAs for human miRNA gene prediction. *Bioinformatics* **25**, 989–995 (2009).
9. Kinsella, R. J. *et al.* Ensembl BioMarts: a hub for data retrieval across taxonomic space. *Database* **2011**, bar030 (2011).
10. ENCODE Project Consortium. An integrated encyclopedia of DNA elements in the human genome. *Nature* **489**, 57–74 (2012).
11. Contrino, S. *et al.* modMine: flexible access to modENCODE data. *Nucleic Acids Res.* **40**, D1082–8 (2012).
12. Zhang, Y. *et al.* Model-based analysis of ChIP-Seq (MACS). *Genome Biol.* **9**, R137 (2008).
